# The Need for Structured Strategies to Improve Stroke Care in a Rural Telestroke Network in Northern New South Wales, Australia: An Observational Study

**DOI:** 10.3389/fneur.2021.645088

**Published:** 2021-04-09

**Authors:** Yumi Tomari Kashida, Carlos Garcia-Esperon, Thomas Lillicrap, Ferdinand Miteff, Pablo Garcia-Bermejo, Shyam Gangadharan, Beng Lim Alvin Chew, William O'Brien, James Evans, Khaled Alanati, Andrew Bivard, Mark Parsons, Jennifer Juhl Majersik, Neil James Spratt, Christopher Levi, The members of Northern NSW Telestroke investigators for this project

**Affiliations:** ^1^School of Medicine and Public Health, The University of Newcastle, Newcastle, NSW, Australia; ^2^Department of Neurology, John Hunter Hospital, Newcastle, NSW, Australia; ^3^Department of Neuroscience, Gosford Hospital, Gosford, NSW, Australia; ^4^Department of Neurology, Melbourne Brain Center at Royal Melbourne Hospital, Parkville, VIC, Australia; ^5^Department of Neurology, Liverpool hospital, Liverpool, NSW, Australia; ^6^Department of Neurology, The University of Utah, Salt Lake City, UT, United States; ^7^Hunter New England Health, Wallsend, NSW, Australia

**Keywords:** telestroke, acute stroke care, multimodal computed tomography, door-to-needle time, thrombolysis, thrombectomy

## Abstract

**Introduction:** A telestroke network in Northern New South Wales, Australia has been developed since 2017. We theorized that the telestroke network development would drive a progressive improvement in stroke care metrics over time.

**Aim:** This study aimed to describe changes in acute stroke workflow metrics over time to determine whether they improved with network experience.

**Methods:** We prospectively collected data of patients assessed by telestroke who received multimodal computed tomography (mCT) and were diagnosed with ischemic stroke or transient ischemic attack from January 2017 to July 2019. The period was divided into two phases (phase 1: January 2017 – October 2018 and phase 2: November 2018 – July 2019). We compared median door-to-call, door-to-image, and door-to-decision time between the two phases.

**Results:** We included 433 patients (243 in phase 1 and 190 in phase 2). Each spoke site treated 1.5–5.2 patients per month. There were Door-to-call time (median 39 in phase 1, 35 min in phase 2, *p* = 0.18), and door-to-decision time (median 81.5 vs. 83 min, *p* = 0.31) were not improved significantly. Similarly, in the reperfusion therapy subgroup, door-to-call time (median 29 vs. 24.5 min, *p* = 0.12) and door-to-decision time (median 70.5 vs. 67.5 min, *p* = 0.75) remained substantially unchanged. Regression analysis showed no association between time in the network and door-to-decision time (coefficient 1.5, *p* = 0.32).

**Conclusion:** In our telestroke network, acute stroke timing metrics did not improve over time. There is the need for targeted education and training focusing on both stroke reperfusion competencies and the technical aspects of telestroke in areas with limited workforce and high turnover.

## Introduction

The increased availability of telestroke in Australia means that physicians from rural areas can consult expert stroke neurologists at comprehensive stroke centers (1). This support leads to an increasing number of patients receiving reperfusion therapies (2–5), being particularly relevant after the recent trials expanding the window for endovascular thrombectomy (EVT) (6, 7) and thrombolysis (8).

Since April 2013, a telestroke network using multimodal computed tomography (mCT - including brain non-contrast CT, CT angiography, and CT perfusion) has been developed in Northern New South Wales, Australia. This network had a progressive expansion, accelerated since 2017, increasing the number of patients assessed *via* telestroke from 48 in 2016 to over 600 in 2019 (9, 10).

In August 2019, as part of a support system for regional and rural stroke care, the clinical trial TACTICS (Trial of Advanced CT Imaging and Combined Education Support for Drip and Ship) was launched. TACTICS aimed to boost access to reperfusion therapies using a multifaceted education and training program targeting opportunities for workflow improvement in regional and rural acute stroke care (ACTRN12619000750189). In this study, we aim to describe the acute stroke treatment timing metrics in our telestroke network following full implementation of telestroke care across the network in 2017 but prior to commencement of the TACTICS trial. We hypothesized there would be trends to improvement in workflows with growth in network experience over time (11, 12).

## Materials and Methods

### Telestroke Network

The network commenced with a single spoke hospital in April 2013, with a second site added in 2014 (13). In 2017, the telestroke network incorporated three new spoke hospitals, finishing its expansion in 2018 with the inclusion of the last hospital. The rural population that the telestroke network covers has increased from 110,000 in 2014 to 300,000 people (13) in 2018, distributed over an area of 143,120 km (slightly larger than England) (2). The John Hunter Hospital (Newcastle) is the comprehensive stroke center (the hub hospital), and sole hospital providing EVT to the area. In our network, all stroke patients except those who require EVT are treated in spoke hospitals.

Regular visits to the spoke sites were performed by the hub team (average of 1–2 per year), and teaching sessions focused on acute stroke pathway were organized (2–3 per year). Immediate feedback after complex cases was regularly given *via* local stroke coordinators.

All six spoke hospitals were equipped with cameras in the Emergency Department and the local radiographers were trained in multimodal CT acquisition. We refer the readers to previous publications, in which our telestroke workflow has been described in detail (9, 10).

### Study Design and Data Collection

Patients assessed by telestroke between January 2017 and July 2019 were included.

Patients from the 2013–2016 period were not included in the analysis because the network expansion and infrastructure deployment were still in progress. During this period just 112 patients were assessed.

We divided the analysis in two phases: Phase 1, from January 2017 to October 2018 and phase 2, from November 2018 to July 2019. Phase 1 was selected to begin at the formalization of the central telestroke network and end once the majority of sites had participated in the network for at least 12 months. We excluded one site that was not initiated telestroke until September 2018 and this site contributed fewer than 20 consults per year. Clinical data was prospectively collected including baseline demographics, National Institute of Health Stroke Scale (NIHSS), mCT characteristics (vessel occlusion status, ischemic core and penumbra volumes), and modified Rankin Scale (mRS) before, and 3 months after, stroke. Large vessel occlusion (LVO) was defined as an occlusion in the proximal segment of the middle cerebral artery (M1 or M2), terminal internal carotid artery, basilar artery or combined intra- and extracranial occlusions. Date and time of stroke onset (or last time seen well), telestroke consult initiation, treatment decision time, and thrombolysis time were also collected. Onset-to-door time was calculated as the difference between stroke onset/last time seen well and Emergency Department (ED) arrival. Door-to-call time was calculated as the difference between patient arrival at the rural hospital and the call to the telestroke neurologist. Door-to-image time was calculated as the difference between patient arrival and mCT initiation. Door-to-decision time was calculated as difference between patient arrival and time when the neurologist notified the spoke team of the plan for the patient (candidate/not candidate for reperfusion therapies). Door-to-needle time was calculated as the difference between patient arrival time and thrombolysis bolus initiation. Patients who suffered a stroke in hospital were excluded from door-to-call, door-to-imaging and door-to-decision analyses. Patients were diagnosed with a stroke mimic by telestroke neurologists if the clinical presentation was atypical for stroke and follow up brain CT/MRI was normal.

### Imaging Protocol

The mCT imaging protocol included brain non-contrast CT, CT angiography, and CT perfusion at baseline and either brain CT or MRI follow-up as per routine clinical practice. All perfusion images were analyzed using MIStar (Apollo Medical Imaging Technology, Melbourne, Australia). Tissue with a delay time >3 s and a relative cerebral blood flow >30% of the contralateral hemisphere was defined as penumbra, and the tissue with a delay time >3 s and a relative cerebral blood flow <30% was defined as ischemic core (14).

### Statistical Analysis

Groups were compared with the independent samples Mann–Whitney *U*-test. For categorical variables, Pearson's Chi squared test or Fisher's exact test were used as appropriate. Statistical significance was set at 0.05. Time metrics were also assessed using a regression model. To examine the effect of telestroke experience, we set treatment timings as a dependent variable and set the time elapsed since January 2017, NIHSS, ED arrival within 4.5 h from the onset, and reperfusion therapy as independent variables, since our previous research showed symptom severity, fast ED arrival and reperfusion therapy related to faster treatment timings (15). Partial missing data from the database are random and the proportion of cases missing data was relatively small, thus we used the case deletion method. All statistical analysis was performed on Stata version 14 (Statacorp, USA). Results are presented as mean ± standard deviation (SD) or median [Interquartile range-IQR].

## Results

**Figure 1:**
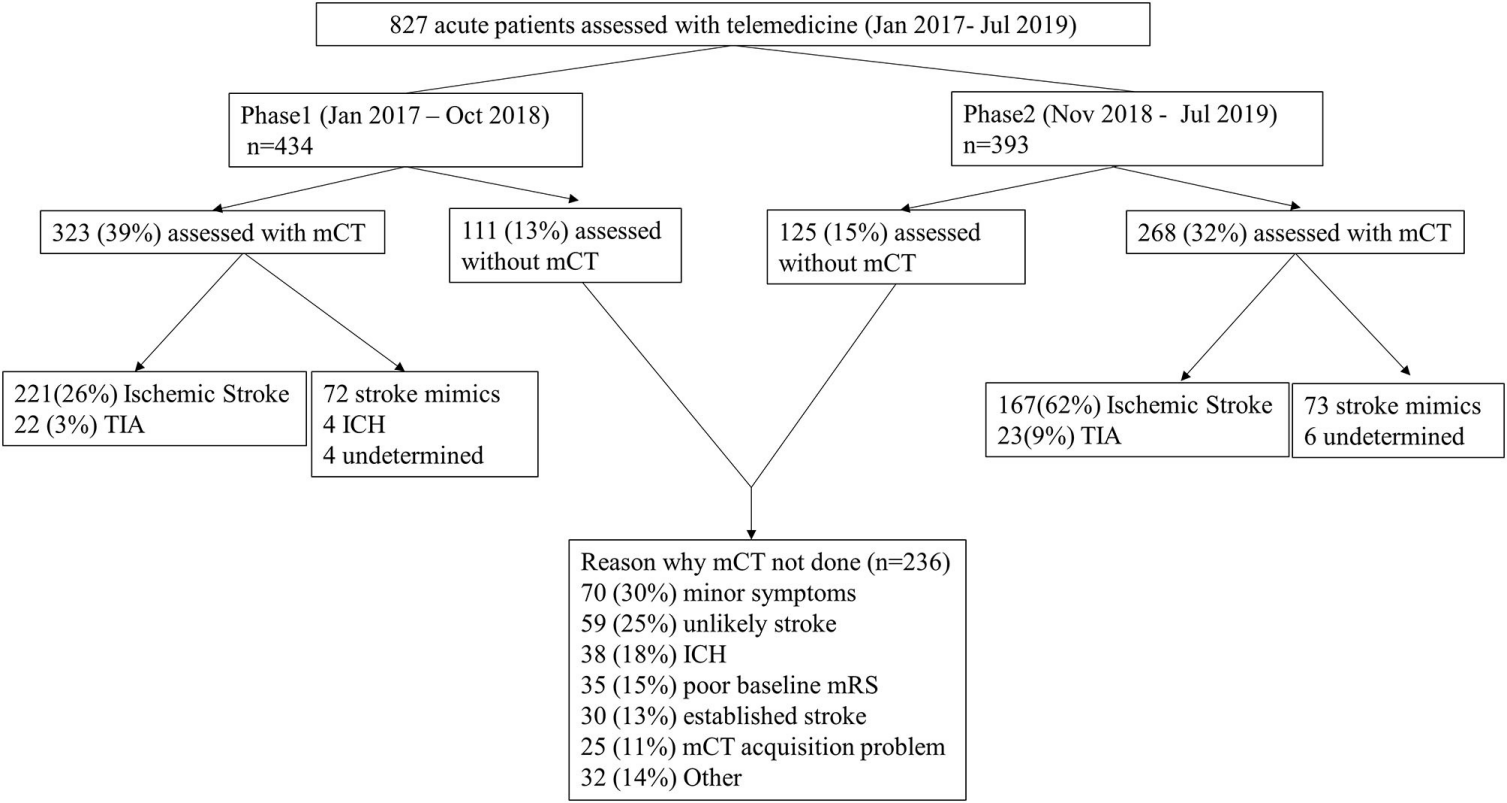
Diagnoses in each phase and the reasons why mCT was not used. mCT, multimodal Computed Tomography (CT); TIA, Transient Ischemic Attack; ICH, Intracranial Hemorrhage; NCCT, non-contrast CT; mRS, modified Rankin Scale.

From January 2017 to July 2019, 827 patients were assessed by the telestroke network, 434 in phase 1 and 393 in phase 2. mCT was performed in 591 patients (71.5%) (323 and 268 patients in phase 1 and 2, respectively). Of those patients receiving mCT, 433 (73.3%) were confirmed to have ischemic stroke or transient ischemic attack (TIA) and 145 (24.5%) were stroke mimics. 236 patients (28.5%) did not undergo urgent mCT for the following reasons: minor symptoms at presentation (73 cases), initial presentation unlikely to be a stroke based on neuro-assessment (59 cases) or presence of hemorrhage in brain NCCT (38 cases) (Figure 1).

The median age of the 433 patients with confirmed ischemic stroke/TIA was 74 years [IQR 64–81] and 277 were male (64%). Most of the patients were independent before their stroke (mRS 0–2, 391 patients, 90.3%). The median baseline NIHSS was 4 [2–9], and 121 patients (28.0%) had a large vessel occlusion. The median penumbra volume was 5 [0–41] ml and the median core volume was 1 [0–7] ml. A total of 109 patients (25.2%) received reperfusion therapies; 74 patients (68.0%) underwent thrombolysis, 58 received EVT (53.2%), and 23 (21.1%) patients underwent combined therapy.

**Figure 2:**
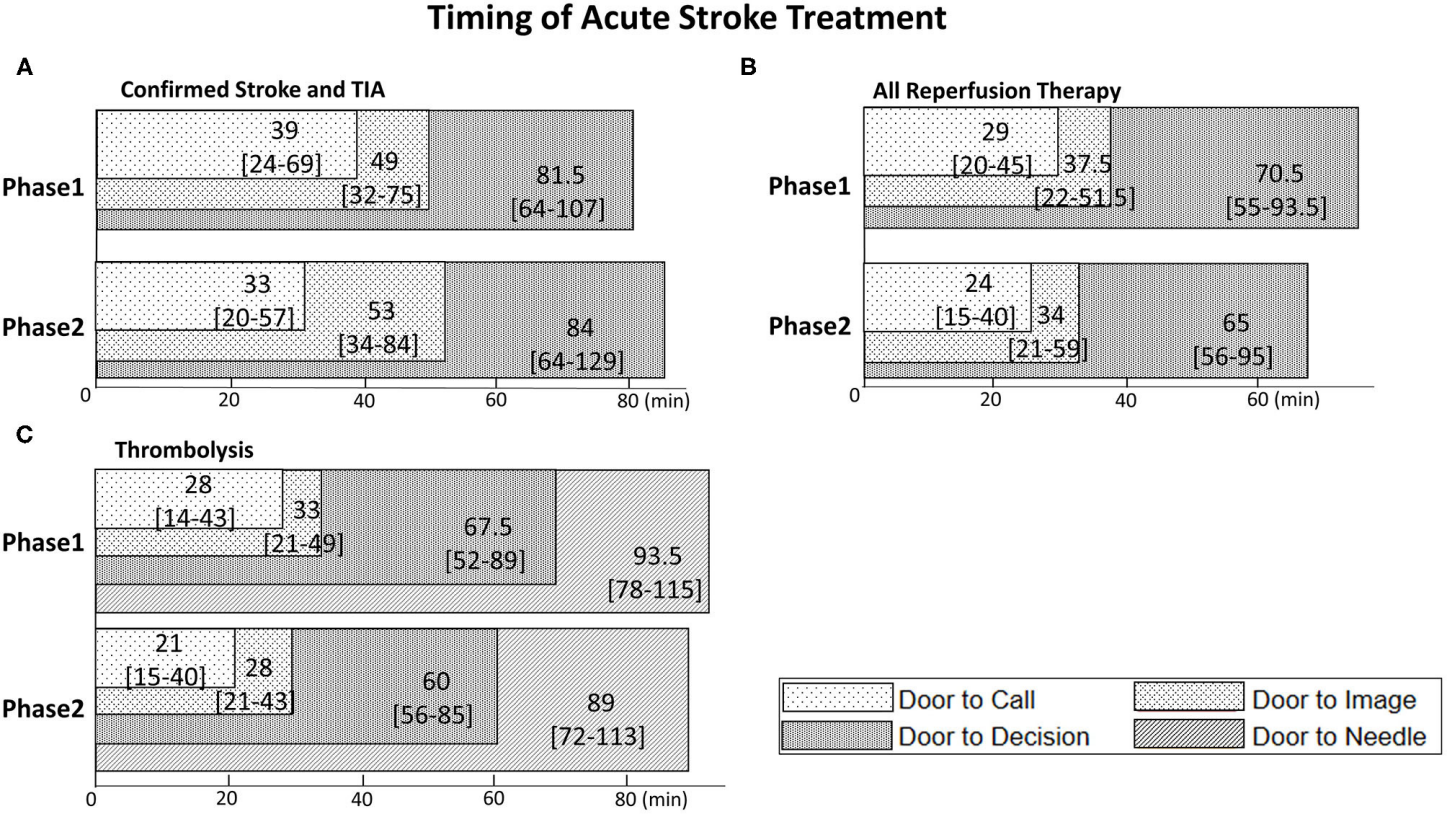
Superimposed bar graphs of Door-to-Call, Door-to-Image, Door-to-Decision, and Door-to-Needle time. Numbers in each bar are median time and [IQR] (min). **(A)** Confirmed ischemic stroke patients and TIA patients in phase 1 and 2. **(B)** Patients who underwent reperfusion therapy. **(C)** Patients who underwent thrombolysis.

As a whole, the median door-to-call time was 36 [IQR 21–63] min, the median door-to-image was 50 [33–77] min, and the median door-to-decision was 82 [64–118] min. There was no significant difference between phases for these metrics. Phase 1 had a median door-to-call of 39 min compared to 35 min in phase 2 (*p* = 0.18), median door-to-image was 49 min in phase 1 and 54 min in phase 2 (*p* = 0.36) and median door-to-decision was 81.5 min in phase 1 and 83 min in phase 2 (*p* = 0.31) (Table 1, Figure 2A). The results did not change even when experience was analyzed based on the date of each individual site's telestroke initiation (data not shown).

**Table 1:** Clinico-radiological characteristics and workflow metrics of ischemic stroke and transient ischemic attack patients assessed with mCT.

	**Total (*n* = 433)**	**Phase 1 (*n* = 243)**	**Phase 2 (*n* = 190)**	***P*-value**
Age, median [IQR]	74 [65–81]	74 [65.5–82]	73 [64–81]	0.28
Sex, male (%)	277 (64)	157 (64.6)	120 (63.2)	0.85
Baseline mRS 0–2 (%)	391 (90.3)	218 (89.7)	173 (91.1)	0.60
NIHSS, median [IQR]	4 [2–9]	4 [2–9]	5 [2–9]	0.63
**Vascular risk factors**, ***n*** **(%)**				
Hypertension	259 (59.8)	156 (64.2)	103 (54.2)	0.04
Hypercholesterolemia	132 (30.5)	71 (29.2)	61 (32.1)	0.52
Type 2 diabetes mellitus	71 (16.4)	40 (16.5)	31 (16.3)	0.97
Prior stroke/TIA	117 (27)	66 (27.2)	51 (26.9)	0.94
Atrial fibrillation	89 (20.6)	42 (17.3)	47 (24.8)	0.05
Ischemic heart disease	89 (20.6)	46 (18.9)	43 (22.6)	0.34
**Multimodal CT characteristics**				
Core, median [IQR] ml	1 [0–7]	1 [0–8]	0 [0–6]	0.38
Penumbra, median [IQR] ml	5 [0–41]	8 [0–52]	4 [0–32]	0.14
Large vessel occlusion, *n* (%)	121 (28)	71 (29.2)	50 (26.3)	0.45
**Reperfusion therapy**				
Any reperfusion therapy	109 (25.2)	68 (28)	41 (21.6)	0.13
Thrombolysis (with or without EVT)	74 (17.1)	52 (21.4)	22 (11.6)	<0.01
EVT (with or without thrombolysis)	58 (13.4)	31 (12.8)	27 (14.2)	0.66
**Time metrics, median [IQR] min**				
Onset-to-Door^*^	127.5 [75–336]	119.5 [73–284]	138 [80.5–440]	0.31
Door-to-Call	36 [21–63]	39 [24–69]	35 [20–59]	0.18
Door-to-Image	50 [33–77]	49 [32–75]	54 [34–85.5]	0.36
Door-to-Decision	82 [64–118]	81.5 [64–107]	83 [64–130]	0.31
Call-to-Decision	43 [30–60]	40 [25–55]	47 [35–62]	<0.01

*mRS, modified Rankin Scale; NIHSS, National Institute of Health Stroke Scale; TIA, Transient Ischemic Attack; EVT, Endovascular thrombectomy*,

* *Missing data: Onset-to-Door time and Door-to-Call/-Image/-Decision, and Call-to-Decision in 22 patients. Door-to-Image time in 7 patients*.

Regression analysis showed no significant association between the time from telestroke network initiation and any stroke metrics (door-to-call, door-to-image, and door-to decision times) except call-to-decision time, which showed 2.0 min increase in delay per 3 months (95% CI 0.6–3.4, *p* < 0.001) (Table 2).

**Table 2:** Estimated effects on treatment timings of the experience, NIHSS, ED arrival within 4.5 h and the use of reperfusion therapy.

**Variable**		**Time since Jan 2017** ** (quarters)**	**NIHSS**	**ED arrival 4.5 h from the onset**	**Reperfusion therapy**	**R^**2**^**
Door-to-Image	β (95% CI)	0.85 (−2.5 to 4.2)	−1.4 (−2.8 to −0.06)	−31.6 (−47.7 to −15.6)	−21.3 (−39.6 to −2.9	0.084
	*P*-value	0.62	0.04	<0.001	0.02	
Door-to-Call	β (95% CI)	−1.1 (−3.8 to 1.6)	−0.7 (−1.8 to 0.4)	−22.7 (−35.3 to −10.1)	−15.9 (−30.6 to −1.43	0.063
	*P*-value	0.31	0.27	<0.001	0.03	
Door-to-Decision	β (95% CI)	1.5 (−1.4 to 4.4)	−1.1 (−2.3 to 0.1)	−25.8 (−39.7 to −11.9)	−17.5 (−33.4 to −1.6	0.081
	*P*-value	0.32	0.08	<0.001	0.03	
Call-to-Decision	β (95% CI)	2.0 (0.6 to 3.4)	−0.5 (−1. to 0.05)	−4.3 (−10.5 to 1.8)	−0.26 (−7.1 to 7.6)	0.04
	*P*-value	0.004	0.08	0.17	0.9	

*NIHSS, National Institute of Health Stroke Scale; ED, emergency department; β, beta coefficient in regression analysis*.

### Reperfusion Therapy Subgroup

Of the 109 patients who received reperfusion therapy, the median age was 73 [64–80] years and 66 were male (60.6%), and most of them (98.2%) were independent at baseline. The baseline NIHSS was 9 [6–15], and 84 (77.1%) had a large vessel occlusion. The baseline characteristics between phase 1 and 2 for these patients were similar, but there was a higher proportion of male sex in phase 1 (69.1% in phase 1 vs. 46.3% in phase 2, *p* = 0.02) and a higher proportion of large vessel occlusion strokes in phase 2 (70.6 vs. 87.8%, *p* = 0.04). There was a significant decrease in the rate of thrombolysis in phase 2 compared to phase 1 (76.5 vs. 53.7%, *p* = 0.01) but an increase in use of EVT (45.6 vs. 65.9%, *p* = 0.04) (Table 3).

**Table 3:** Clinico-radiological characteristics and workflow metrics of patients who underwent reperfusion therapy.

	**Total (*n* = 109)**	**Phase 1 (*n* = 68)**	**Phase 2 (*n* = 41)**	***P*-value**
Age median [IQR]	73 [64–80]	73 [64–80]	71 [61–77]	0.4
Sex male (%)	66 (60.6)	47 (69.1)	19 (46.3)	0.02
Baseline mRS 0–2(%)	107 (98.2)	67 (76.5)	40 (97.6)	0.73
NIHSS median [IQR]	9 [6–15]	9 [6–14]	10 [6–17]	0.37
**Multimodal CT characteristics**				
Core, median [IQR] ml	7 [2–18]	6 [1–16]	9 [4–19]	0.2
Penumbra, median [IQR] ml	61 [34−94.5]	62 [35–91]	54 [34–107]	0.98
Large vessel occlusion, *n* (%)	84 (77.1)	48 (70.6)	36 (87.8)	0.04
**Reperfusion therapy**				
Thrombolysis	74 (62)	52 (76.5)	22 (53.7)	0.01
EVT	58 (48.7)	31 (45.6)	27 (65.9)	0.04
Combination	23 (19.3)	15 (22.1)	8 (19.5)	0.75
Time course, median [IQR] min^*^				
Onset-to-Door	93.5 [65–158]	88 [66–133]	113 [60–199]	0.59
Door-to-Call	26.5 [17–43]	29 [20–45]	24.5 [15–40]	0.12
Door-to-Image	35 [21–52]	37.5 [22–51.5]	34.5 [21–59]	0.87
Door-to-Decision	70 [55–95]	70.5 [55–93.5]	67.5 [56–95]	0.82
Call-to-Decision	41 [32–51]	37.5 [25–49]	47 [34–63]	0.02

*mRS, modified Rankin Scale; NIHSS, National Health Institute of Health Scale; TIA, Transient Ischemic Attack; EVT, Endovascular therapy*.

* *Missing data: Onset-to-Door time, Door-to-Call time, and Call-to-Decision time in three patients. Door-to-Image time and Door-to-Decision time in one patient*.

The median door-to-call time was 26.5 min [17–43], median door-to-image was 35 min [21–52], and median door-to-decision was 70 min [55–95]. All these metrics were slightly shorter in phase 2, but none of the differences was significant. Door-to-call was 29 min in phase 1 vs. 24.5 in phase 2 (*p* = 0.12), door-to-image 37.5 min vs. 34.5 min (*p* = 0.87) and door-to-decision was 70.5 min in phase 1 and 67.5 min in phase 2 (*p* = 0.82); see Table 3 and Figure 2B.

In the thrombolysed subgroup, door-to-needle time were slightly shorter in phase 2 (93.5 min in phase 1 vs. 89 min in phase 2, *p* = 0.5) but this difference was not significant. No difference was found in any of the other metrics (Figure 2C).

Regression analysis demonstrated no significant association between time since the telestroke network initiation and door-to-needle (Adjusted β = 1.1 min, *p* = 0.62) or decision-to-needle times (Adjusted β =1.2 min, *p* = 0.15).

## Discussion

In this study, we described the temporal trends in acute stroke workflow metrics in our telestroke network. We noted no significant improvements in process of care metrics over a period of 2.5 years. Although, some publications suggest that participation in telestroke networks improves treatment timings in acute stroke (11, 12), this was not reflected in the data from our network. Door-to-call time slightly improved in phase 2 compared to phase 1 (from 39 to 35 min), however, door-to-image and door-to-decision times were slightly extended.

There are several possible explanations for this slight increase in time metrics in our telestroke network. Firstly, there is a limited number of acute stroke patients that each spoke hospitals' physicians could see (1.5–5.2 stroke patients per month in the study period). In addition, the rural hospitals suffer from workforce shortages and a high turnover across all career stages, from specialists to trainees (16). Therefore, clinical experience does not concentrate within a specific team but disseminates. Staff experience is an essential factor to improve acute stroke quality, and there is a report that showed center volume had a strong effect on shortened Door-to-Needle time (17). The hub stroke team has provided regular visits and feedback through stroke coordinators at the spoke sites, nevertheless, our result showed that this support was not enough to improve acute stroke timing metrics for spoke hospitals in our telestroke network. Therefore, there is a need for structured strategies including stroke-specific education and training, and a system that encourages and motivates health professionals in spoke hospitals. Rural settings often face well-recognized barriers to system change, including facility leadership support, competing demands, stakeholder resistance, resource, and technical support constraints (18–21). We speculate that a lack of improvement in acute stroke time metrics might be ascribed to these disadvantages.

Previous studies have indicated that telestroke networks can successfully improve their performance level with appropriate initiatives to improve the quality of acute stroke care (22, 23). Aiming to improve acute stroke workflows and times to reperfusion, a trial across the network with a focus in multifaceted education and support for workflow re-design (the TACTICS trial), is now underway. The intervention includes teleconferences within the network and reviews of current acute stroke workflow to identify barriers and provide prioritized solutions and strategies as well as stroke-specific education to health professionals (ACTRN12619000750189). The comprehensive stroke center team collaborate with the network hospitals by simulating action planning and ongoing clinical practice improvement activities.

Besides reperfusion therapy, all patients who suffer stroke can benefit from specialized stroke-unit care, and telestroke networks can play a role in this (24). Each spoke site has a stroke unit that provides care to all stroke patients admitted to that site, but the function of the telestroke network is currently limited to the provision of acute reperfusion therapy. Establishing a system that supports and educates health professionals in spoke hospitals regarding general acute stroke care through telestroke remains a long-term goal.

Time metrics in our telestroke network are comparatively long, considering the guideline recommendation (25). Workforce shortages (emergency teams must treat multiple patients simultaneously), inefficient workflows (absence of pre-notification from paramedics, telestroke activation call after initial imaging), and the infrequent use of acute reperfusion therapies by health professionals in spoke hospitals all contribute to the slow time metrics (Supplementary Table 1). In addition, our telestroke network uses mCT as the standard image for acute stroke care. We acknowledge that mCT adds 10–13 min to the door-to-decision/needle times in the acute phase, but CT perfusion is required to select candidates for EVT in the extended window and provides invaluable information regarding tissue prognosis in the standard window. In a geographically disparate network like ours, where transfers can take over 5 h and transport assets are extremely limited, careful triage to avoid unnecessary transfers is essential (9, 10).

There are some limitations in this study, most importantly the small number of patients treated. The requirement for patients to undergo mCT to be included in this analysis introduces a potential bias, as stroke patients who did not undergo mCT due to technical failures were excluded. However, the occurrence rate of the technical failures was low (3% of the whole sample in this study) and random in nature, so those patients are unlikely to affect the result in either a positive or negative direction. Lastly, the generalizability of the study is limited, and it may only be applicable to other rural telestroke networks with similar structure and limited workforce. Conversely, strengths include the fact that all the data were collected prospectively, and various analyses of the timing data consistently show no significant difference between groups, including the regression analysis which did not require any arbitrary time division. While this study may not be generalizable to all telestroke networks, the problems of limited experience in spoke hospitals, high staff turnover and work-force shortages are common problems in rural areas both in Australia and overseas (26, 27).

In conclusion, our study shows that in our telestroke network, acute stroke timing metrics did not improve over time. There is the need for targeted education and training focusing on both stroke reperfusion competencies, and the technical aspects of telestroke in areas with limited workforce and high turnover.

## Data Availability Statement

The raw data supporting the conclusions of this article will be made available by the authors, without undue reservation.

## Ethics Statement

The studies involving human participants were reviewed and approved by the Hunter New England Health Ethics Committee. Written informed consent for participation was not required for this study in accordance with the national legislation and the institutional requirements.

## Author Contributions

CG-E, NS, and CL conceived the present study. CG-E, FM, PG-B, SG, BC, WO'B, JE, and KA acquired the data, interpreted imaging data, and reviewed the manuscript. TL analyzed and interpreted the data and edited the manuscript. YK and CG-E analyzed the data and drafted and edited the manuscript. AB, MP, JM, NS, and CL supervised the study and critically reviewed the manuscript. All authors read and approved the final manuscript.

## Conflict of Interest

The authors declare that the research was conducted in the absence of any commercial or financial relationships that could be construed as a potential conflict of interest.
