# Supplementary material for: The Need for Structured Strategies to Improve Stroke Care in a Rural Telestroke Network in Northern New South Wales, Australia: An Observational Study
Source: Front Neurol. 2021 Apr 9;12:645088. doi: 10.3389/fneur.2021.645088 (PMC8064411; doi:10.3389/fneur.2021.645088)
Supplement: Supplementary file 1 [file Table_1.PDF]

Supplementary Table S1. Potential factors of the delay in acute time metrics

Time metrics	Door-to-Call/image	Image-to-Decision	Decision-to-Needle
Potential factors of the delay	• The lack of prenotification from paramedics across all the rural sites<br>• Delay in activation of telestroke consult due to the lack of stroke-dedicated teams<br>• Workforce shortage (e.g. no stroke dedicated teams at ED, no radiographers on site after-hours)	• Activation of telestroke consult after image<br>• Multimodal CT scan analysis time (10-15min)<br>• Image transfer to the web-server (5-10min)	• Limited experience in reperfusion therapy<br>• The workforce shortage: one or two health professionals have to manage the necessary work-up and preparation for reperfusion therapy (e.g. acquiring consent from family, drug preparation and administration)
