# Supplementary material for: The Need for Structured Strategies to Improve Stroke Care in a Rural Telestroke Network in Northern New South Wales, Australia: An Observational Study
Source: Front Neurol. 2021 Apr 9;12:645088. doi: 10.3389/fneur.2021.645088 (PMC8064411; doi:10.3389/fneur.2021.645088)
Supplement: Supplementary file 2 [file Data_Sheet_1.PDF]

Northern NSW Telestroke investigators

Ms. Rachel Peake, Dr. James Hughes, Dr. Lisa Dark, Dr. Nick Ryan, Dr. Matt Shepherd, Dr. Osama Ali, Dr. Dewald Behrens, Ms. Fiona Minett, Ms. Jaclyn Birnie, Ms. Amanda Buzio, Dr. Iain Bruce, Dr. Alan Tankel, Ms. Kim Parrey, Dr. Matthew Kinchington, Dr. Elizabeth Pepper, Dr. Andre Loiselle, Dr. Thomas Wellings, Ms. Michelle Russell, Ms. Angela Royan, Mr. Brett Roworth
